# Povidone-iodine hand wash and hand rub products demonstrated excellent *in vitro* virucidal efficacy against Ebola virus and modified vaccinia virus Ankara, the new European test virus for enveloped viruses

**DOI:** 10.1186/s12879-015-1111-9

**Published:** 2015-09-17

**Authors:** Maren Eggers, Markus Eickmann, Katharina Kowalski, Juergen Zorn, Karen Reimer

**Affiliations:** Labor Prof. Gisela Enders MVZ GbR and the Institute of Virology, Infectious Diseases and Epidemiology e.V., Stuttgart, Rosenbergstr. 85, 70193 Stuttgart, Germany; Institute for Virology, Philipps University of Marburg, Marburg, Germany; Mundipharma Research GmbH & Co.KG, Höhenstrasse 10, 65549 Limburg, Lahn Germany; University Witten/Herdecke gGmbH, Witten, Germany

## Abstract

**Background:**

The recent Ebola virus (EBOV) epidemic highlights the need for efficacious virucidal products to help prevent infection and limit the spread of Ebola virus disease. However, there is limited data on the efficacy of virucidal products against EBOV, because the virus has a high biosafety level and is only available in a few laboratories worldwide.

The virucidal efficacy of antiseptics and disinfectants can be determined using the European Standard EN14476:2013/FprA1:2015. Modified vaccinia virus Ankara (MVA) was introduced in 2014 as a reference virus for the claim ‘virucidal active against enveloped viruses for hygienic hand rub and hand wash’. For EBOV, also an enveloped virus, the suitability of MVA as a surrogate needs to be proven.

The aim of this study was to test the *in vitro* efficacy of four povidone iodine (PVP-I) formulations against EBOV: 4 % PVP-I skin cleanser; 7.5 % PVP-I surgical scrub; 10 % PVP-I solution; and 3.2 % PVP-I and 78 % alcohol solution. The formulations were tested with MVA to define the test conditions, and as a secondary objective the suitability of MVA as a surrogate for enveloped viruses like EBOV was assessed.

**Methods:**

According to EN14476, a standard suspension test was used for MVA. Large-volume plating was used for EBOV to increase test sensitivity and exclude potential after-effects. All products were tested under clean (0.3 g/L BSA) and dirty (3.0 g/L BSA + 3.0 mL/L erythrocytes) conditions with MVA for 15, 30, and 60 s. The concentration-contact time values obtained with MVA were verified for EBOV.

**Results:**

Viral titres of MVA and EBOV were reduced by >99.99 % to >99.999 % under clean and dirty conditions after application of the test products for 15 seconds.

**Conclusions:**

All products showed excellent virucidal efficacy against EBOV, demonstrating the important role PVP-I can play in helping to prevent and limit the spread of Ebola virus disease. The efficacy against both test viruses after 15 s is helpful information for the implementation of guidance for people potentially exposed to EBOV, and confirms the excellent virucidal efficacy of PVP-I against enveloped viruses. MVA was found to be a suitable surrogate for enveloped viruses like EBOV.

## Background

In December 2013, the Ebola virus (EBOV) epidemic began in Guinea, and on March 23, 2014, the Ebola virus outbreak was officially communicated by the World Health Organization (WHO). This outbreak in West Africa (mostly affecting Guinea, Sierra Leone and Liberia) has been the largest and most complex outbreak since the virus was discovered.

EBOV is spread mainly through contact with body fluids of symptomatic patients or contaminated surfaces [1], so healthcare workers and the family and friends in close contact with Ebola patients are at the highest risk of becoming infected and/or further spreading the virus [2]. EBOV disease has a fatality rate of up to 90 %, and there is no specific antiviral treatment or vaccine currently available [1], although Phase I clinical trials with the most advanced Ebola vaccine candidates started in autumn 2014 [3–5]. Current treatment includes general care to support vital organ functions, including fluid replacement therapy, kidney dialysis, blood transfusions and plasma replacement therapy [2]. In the absence of EBOV-specific treatments, efficacious disinfectant and antiseptic products are useful to help prevent the spread of infection [6]. In addition, hygiene measures, such as wearing gloves for any contact with blood and body fluids, medical masks and goggles or face shields have been identified as very important to protect against EBOV transmission.

Considering that EBOV is a deadly threat, it is clear that only chemical disinfectants with proven virucidal efficacy can be used. This can be achieved by ensuring that disinfectants pass a virucidal activity test performed in compliance with good laboratory practice and country-specific standards. In Europe, EN14476 [7] describes the standard for determining virucidal activity, which involves three non-enveloped viruses: poliovirus type 1 LSc-2ab, adenovirus type 5 strain (AdV-5) Adenoid 75, and murine norovirus, but until recently, no enveloped virus. Interestingly, for several years the German guideline for virucidal testing [8] has included tests of disinfectants against enveloped viruses including MVA and bovine viral diarrhoea virus (BVDV), allowing products that are effective against enveloped viruses to be labelled as having a ‘limited spectrum of virucidal activity’.

Although enveloped viruses are deemed to be more susceptible to disinfectants, they may react differently than non-enveloped viruses with regard to the concentration and required application time of the active ingredient. Therefore, it is necessary to test an enveloped model virus for the claim ‘virucidal active against enveloped viruses’. Despite the usual role of enveloped viruses as blood-borne pathogens, e.g. human immunodeficiency, hepatitis B and hepatitis C viruses, they are also responsible for severe outbreaks including severe acute respiratory syndrome (SARS), Middle East respiratory syndrome coronavirus (MERS-Co), influenza pandemic, and hemorrhagic fever viruses such as EBOV.

The CEN/TC 216/WG1 committee, which establishes standardised European testing methods and requirements for the antimicrobial efficacy of chemical disinfectants and antiseptics, intended to implement a new test virus to assess antimicrobial efficacy against all enveloped test viruses. The choice of model viruses is of great importance when establishing such a standard test method. The requirements for model viruses include high resistance to disinfectants and drying, combined with simple virus propagation in cell culture (e.g. growing in high titres). The experts in WG1 suggested the enveloped virus modified vaccinia virus Ankara (MVA) as the reference virus for the claim ‘virucidal active against enveloped viruses for hygienic hand rub and hand wash’. This virus has been shown to produce similar results to vaccinia virus strain Elstree in virucidal testing [9], and was chosen because of its low biosafety level that means it does not pose any hazard to employees performing the tests [10], its known environmental stability and its practicability for laboratory use [11, 12]. In contrast, EBOV is a lethal virus and requires the highest biosafety levels for any investigation. Therefore, it is only available for testing in a few laboratories worldwide.

Povidone iodine (PVP-I) has been known for a long time as a broad spectrum microbicide against bacteria, fungi, protozoans and viruses [13]. In a water-soluble complex, the elemental iodine is bound to the carrier polyvinylpyrrolidone. In an aqueous medium, a chemical equilibrium develops to release the active antimicrobial agent iodine, while the complex-linked iodine builds a reservoir for delivery [14]. Thereby, its microbicidal activity is maintained but the cytotoxic effects of high concentrations of iodine are reduced [14]. The oxidative potency of PVP-I enables the iodine released to react rapidly with functional groups of amino acids and nucleotides, as well as with double bonds of fatty acids, resulting in a manifold destruction of various structures and enzymes of microbes and viruses. The development of resistance mechanisms against the very broad oxidative attack appears almost impossible [13, 15].

Compelling *in vitro* data for the general virucidal activity of PVP-I is available, demonstrating efficacy against enveloped viruses such as mumps, herpes simplex, rubella, measles, influenza, human immunodeficiency and corona viruses, and non-enveloped viruses including adeno-, rota-, polio-, coxsackie- and rhinovirus [16–23].

This study investigated the *in vitro* efficacy of four PVP-I hand wash and hand rub products against EBOV. In addition, we explored whether MVA would be a suitable test virus to assess virucidal activity against EBOV, which is also an enveloped virus, by comparing the *in vitro* virucidal efficacy of the PVP-I formulations against MVA with their efficacy against EBOV.

## Methods

### Virucidal products tested

Four PVP-I antiseptic products were tested in this study. These included 4 % PVP-I skin cleanser, 7.5 % PVP-I surgical scrub and 10 % PVP-I solution, all with the brand name Betadine, manufactured by Mundipharma (Limburg, Germany), and a 3.2 % PVP-I/alcohol solution containing 78 % alcohol (2-propanol and ethanol), with the brand name Betaseptic, also manufactured by Mundipharma.

### Propagation of the test virus

#### MVA

Test virus suspensions were prepared by infecting susceptible cells with MVA from the Institute of Animal Hygiene and Veterinary Public Health at the University of Leipzig. BHK-21 cells, a cell line established from fibroblasts of newborn hamster kidneys were used for virus cultivation and the suspension test. After virus inoculation of the cells, the supernatant was replaced by minimum essential medium (MEM, Biochrom AG, Germany) with 2 % foetal calf serum (FCS, Sigma-Aldrich, Germany). The host cells (Collection of Cell Lines in Veterinary Medicine [CCLV], Friedrich Loeffler Institute, Germany) were cultivated at 37 °C in a humid atmosphere under 5.0 % CO_2_. For the virus cultivation, confluent monolayers with a maximum age of 2 days were used. The cells were incubated at 37 °C until 70–95 % of the cells exhibited a cytopathic effect (approximately 7 days). The cells were frozen and thawed twice, followed by centrifugation at 1,900 g for 10 min. The virus titre was up-scaled by ultra centrifugation at 4 °C at 53,900 g for 2.5 h. The pellet was resuspended in 2 mL medium and aliquots of the virus suspension were stored at -70 °C.

#### EBOV

EBOV strain Zaire from CDC, Atlanta was used as the test virus. Vero E6 cells (ATCC® CRL-1586™) a cell line established from *Cercopithecus aethiops* kidneys, were used for virus cultivation and the suspension test. The host cells were cultivated at 37 °C in a humid atmosphere under 5.0 % CO_2_. The cells were fed with Dulbecco’s Minimum Essential Medium (D-MEM) supplemented with heat-inactivated FCS and non-essential amino acids. For the virus cultivation, confluent monolayers with a maximum age of 2 days were used. Cell debris was separated by low speed centrifugation at 1,900 g for 10 min. Aliquots of the virus suspension were stored at −70 °C or in liquid nitrogen.

### Determination of cytotoxicity

When using cell culture to evaluate disinfectant activity on viruses, the target cells are often very sensitive to the active ingredient. However this cytotoxic effect seen *in vitro* does not affect the way the products behave *in vivo*. The cytotoxicity of test products in disinfectant testing is commonly overcome by dilution of the virus-disinfectant mixture. In order to determine cytotoxicity, the test products were serially diluted ten-fold in MEM up to a dilution of 10^−8^. One part by volume of water of standardised hardness (instead of virus suspension) was mixed with one part by volume of organic load [clean conditions (0.3 g/l bovine serum albumin; BSA) or dirty conditions (3.0 g/L BSA + 3.0 mL/L erythrocytes)] and eight parts by volume of the test product. Aliquots of 100 μL from each test product at each dilution were then inoculated into six wells of a 96-well microtitre plate containing 200 μL cell suspension BHK-21 cells. The cell cultures were observed for cytotoxic effects for the same incubation time as was later used for the suspension tests.

### Inactivation assay using quantitative suspension test for MVA

Tests were carried out once, in accordance with EN14476:2013/FprA1:2015 at 20 °C ± 1 °C [7]. One part by volume of MVA virus suspension (titre of at least 10^7^–10^8^ tissue culture infectious dose 50 % [TCID_50_]/mL) and one part by volume of the organic load were mixed with eight parts by volume of the PVP-I hand wash or hand rub product. The test products were examined undiluted and as 1:10 and 1:100 solutions. After the specified contact time (15 s, 30 s and 60 s), the virucidal activity was immediately suppressed by dilution with nine volumes of ice-cold medium (MEM + 2.0 % FCS) and without delay the assay was serially diluted 10-fold. Due to the immediate titration, no after-effect of the product could occur. Infectivity was determined by means of end point dilution titration in microtitre plates. Aliquots of 100 μL from each dilution were placed in six wells of a sterile polystyrene flat-bottomed 96-well microtitre plate containing 200 μL BHK-21 cells. Cultures were observed for cytopathic effects after eight days of inoculation. For the virus control, doubly distilled water was applied instead of the test product. All tests were performed under clean conditions (0.3 g/L BSA) and dirty conditions (3.0 g/L BSA + 3.0 mL/L erythrocytes) as interfering substance.

The virus titres were determined using the Spearman-Kärber method [24, 25] and expressed as TCID_50_/mL, including standard deviation. Titre reduction is presented as the difference between the virus titre after contact with the test product and the control virus titre. This difference is given as a reduction factor including its 95 % confidence interval. A reduction in virus titre of ≥4 log_10_ (corresponding to an inactivation of ≥99.99 %) was regarded as evidence of sufficient virucidal activity. The calculation was performed according to EN14476 [7].

### Inactivation assay using large volume plating (LVP) method for verification of concentration-contact time values with EBOV

Usually, only low titres of EBOV can be harvested in cell culture, resulting in a range of 5.00 to 7.70 log_10_ TCID_50_/mL. To demonstrate at least a 4 log_10_ reduction in virus titre, it is necessary for test mixtures containing low virus titres to undergo detoxification by molecular sieving, or to use a more sensitive assay such as LVP [8]. Molecular sieving with Microspin S 400 HR columns is not the method of choice for EBOV because the process reduces the titres of these large, filamentous viruses by 1.00 to 2.00 log_10_. In LVP, a high volume of the lowest apparently non-cytotoxic dilution of the inactivation assay test mixture is added to the detector cell line and the cultures are monitored for virus-specific effects. Using this method, larger reductions in virus titre can be shown even at lower viral loads.

**Fig. 1:**
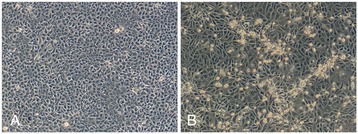
Uninfected and EBOV infected cells. **a** Vero E6 control cells; **b** Viral cytopathic effects seen after infection of Vero E6 cells with EBOV

The inactivation tests were conducted once at 20 °C ± 1 °C. One part EBOV suspension (100 μL) was mixed with 100 μL of either 0.3 g/L BSA (clean conditions) or 3.0 g/L BSA + 3.0 mL/L erythrocytes (dirty conditions) as the interfering substance. The virus-protein mixture was added to 8 parts (800 μL) of the 1:10 diluted test product, with the exception of 4 % PVP-I skin cleanser which was diluted to 1:100. After a contact time of 15 s, 600 μL of the test mixture for the 10 % PVP-I solution, 7.5 % PVP-I surgical scrub, and 3.2 % PVP-I/alcohol solution were added to 57.0 mL ice-cold medium resulting in a 1:96 dilution of the test product. For the 4 % PVP-I skin cleanser, 120 μL of the test mixture was added to 57.6 mL which corresponds to a 1:481 dilution. After dilution, the samples were added in 200 μL aliquots to microtitre plates (288 wells) containing the indicator cells in 100 μL cell culture medium. The cells were cultivated for an incubation period of 6 days, and inspected microscopically for virus foci (virus-induced changes in cell morphology) (Fig. 1). The viral titre was calculated as follows:

### Statistical methods

#### If no virus is observed

The number of infectious virus particles is determined by the Poisson distribution according to the CPMP/ICH/295/95 guideline [26] using the following formula:

$$ \mathbf{c} = \mathbf{\ln}\ \mathbf{p}/\mathbf{\hbox{-}}\mathbf{v} $$

(“c” concentration of viruses in the test mixture; “p“denoting the 95 % probability to detect virus, “v” is the plated volume where “v” is to be << “V” (total volume)).

#### If a low number of viruses is detected

The most probable average number of TCID_50_ can be calculated by the use of the following formula, which is derived from the Taylor series:

$$ Titer/ ml=\frac{D}{V_w}*\left(- \ln \frac{n-{n}_p}{n}\right) $$

(“c” concentration of viruses in the test mixture, “D” dilution factor of prediluted sample, “p“denoting the 95 % probability to detect virus, “V_w_” is the plated volume per well, “n” number of inoculated wells, and “n_p_” is the number of successfully infected wells).

However, one TCID_50_ is equivalent to 0.69 infectious virus particles because the natural logarithm of a 50 % likelihood (p = 0.5) is 0.69 or ln(0.5) = 0.69. This implies that at least 69 trials are necessary to successfully infect 50 % of 100 wells. Therefore this factor is needed to calculate the TCID_50_. In addition, if the Poisson formula is used, the dilution factor of the prediluted sample should be considered.

Using LVP, the lowest apparently non-cytotoxic dilution of the test mixture is added to ice-cold medium after the specified contact time, immediately suppressing the virucidal effects of the test product. This makes it a more precise method for very short incubation times because after-effects of the test product on the sample can be excluded, compared to the endpoint titration Spearman-Kärber method. With this method, it might sometimes be difficult to carry out the necessary pipetting quickly enough, particularly while wearing a BSL-4 protective suit when working with EBOV. In addition, the detection of residual virus can be improved by the testing of a large sample volume. In cases where there are small numbers of virus particles present, the Poisson formula used to calculate virus titre is more precise than the Spearman-Kärber formula.

## Results

### Determination of the PVP-I kinetics with low and high protein load using the new European test virus for virucidal efficacy against enveloped viruses (MVA)

The test concentrations and contact periods were chosen in order to observe the point at which each test preparation produced efficient virus inactivation.

To demonstrate virucidal efficacy, disinfectant and antiseptic products are required to produce a log_10_ reduction in virus titre of at least 4 [7]. The log_10_ reduction factors produced by each test product under clean and dirty conditions at each time point are shown in Table 1.

**Table 1:** Virucidal activity of 4 different PVP-I preparations against MVA

Test product	Dilution	Log_10_ reduction factor (95 % confidence interval, where applicable)					
		Clean conditions			Dirty conditions		
		Application time			Application time		
		15 s	30 s	60 s	15 s	30 s	60 s
PVP-I solution (10 g/L available iodine)	Undiluted (10 g/L)	**≥4.00**	**≥4.00**	**≥4.00**	**≥4.17**	**≥4.17**	**≥4.17**
	1:10 (1 g/L)	**≥5.67**	**≥5.67**	**≥5.67**	**≥5.50**	**≥5.50**	**≥5.50**
	1:100 (0.1 g/L)	**4.33** (±0.63)	**5.67** (±0.60)	**≥6.33**	2.83 (±0.54)	3.50 (±0.65)	3.50 (±0.54)
PVP-I surgical scrub (7.5 g/L available iodine)	Undiluted (7.5 g/L)	**≥4.00**	**≥4.00**	**≥4.00**	**≥4.17**	**≥4.17**	**≥4.17**
	1:10 (0.75 g/L)	**≥5.50**	**≥5.50**	**≥5.50**	**≥5.67**	**≥5.67**	**≥5.67**
	1:100 (0.075 g/L)	3.83 (±0.65)	**4.17** (±0.58)	**4.50** (±0.58)	1.00 (±0.70)	1.67 (±0.70)	1.83 (±0.71)
PVP-I skin cleanser (4 g/L available iodine)	Undiluted (4 g/L)	**≥4.17**	**≥4.17**	**≥4.17**	**≥4.00**	**≥4.00**	**≥4.00**
	1:10 (0.4 g/L)	**4.50** (±0.54)	**≥4.67**	**≥4.67**	**4.33** (±0.56)	**≥4.50**	**≥4.50**
	1:100 (0.04 g/L)	3.33 (±0.56)	3.67 (±0.47)	3.67 (±0.47)	0.33 (±0.56)	1.00 (±0.63)	1.00 (±0.63)
PVP-I/alcohol solution (3.2 g/L available iodine)	Undiluted (3.2 g/L)	**≥5.67**	**≥5.67**	**≥5.67**	**≥5.67**	**≥5.67**	**≥5.67**
	1:10 (0.32 g/L)	**5.50** (±0.54)	**6.33** (±0.56)	**6.33** (±0.56)	3.00 (±0.63)	**4.00** (±0.47)	**4.83** (±0.33)
	1:100 (0.032 g/L)	1.83 (±0.54)	2.00 (±0.60)	2.50 (±0.54)	−0.17 (±0.68)	0.00 (±0.60)	0.17 (±0.54)

Clean conditions: 0.3 g/L bovine serum albumin (BSA); dirty conditions: 3.0 g/L BSA + 3.0 mL/L erythrocytes. Results in bold indicate virucidal activity (≥4 log_10_ reduction in viral titre)

Viral titres of MVA were reduced by >99.99 % to >99.999 % under clean and dirty conditions after application of undiluted and 1:10 dilutions of 10 % PVP-I solution, 7.5 % PVP-I surgical scrub and 4 % PVP-I skin cleanser for as little as 15 s. The 3.2 % PVP-I/alcohol solution had a similar effect, except under dirty conditions at the 1:10 dilution for 15 s, when the reduction factor was 3 (99.9 % reduction).

Under both clean and dirty conditions, a concentration-dependent virucidal activity was seen with the PVP-I only products (Table 1). Under dirty conditions, the minimum concentration of PVP-I needed for a 4 log_10_ reduction of MVA was 5.3 times higher at a 30 s contact time (0.4 g/L versus 0.075 g/L) and 4 times higher at a 15 s contact time (0.4 g/L versus 0.1 g/L), than under clean conditions (Table 1).

### Verification of the concentration-contact time relationship for EBOV

**Fig. 2:**
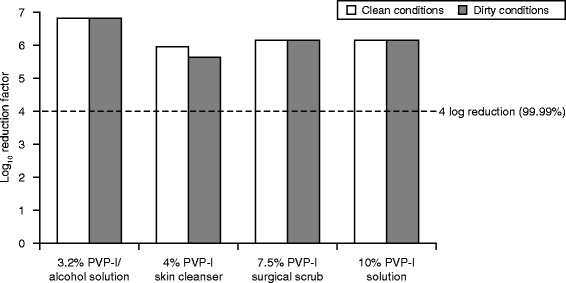
Virucidal activity of four different PVP-I preparations against EBOV strain Zaire. Virucidal activity of four different PVP-I preparations against EBOV strain Zaire after 15 s exposure time

The titres of EBOV present in the control samples were comparable between the test runs, with values around 7 log_10_ TCID_50_/mL. All of the PVP-I preparations tested reduced the EBOV viral titres by a log_10_ reduction factor of between 5.66 and 6.84 after 15 s (Fig. 2). This corresponds to a reduction in EBOV viral titre of >99.999 % for all products tested.

## Discussion

### MVA as a surrogate test virus for EBOV

Until recently, the European guidelines were lacking an enveloped test virus for use when evaluating hygienic hand rub and hand wash. Due to the recent Ebola outbreak, MVA was introduced as a reference virus for all enveloped viruses. However, there was still uncertainty about whether this surrogate would be suitable for dangerous viruses such as EBOV. In this study, we investigated the antiviral efficacy of PVP-I against MVA and then verified the concentration and contact time findings with EBOV. We found that PVP-I was as effective against EBOV as MVA with similar concentration and contact time parameters. In fact, when comparing the results with the 4 % PVP-I skin cleanser, which was pre-diluted to 1:100 for the EBOV test, MVA was even more resistant to PVP-I under dirty conditions than EBOV. Our results suggest that MVA is a suitable surrogate test virus that can be safely used for testing the virucidal efficacy of antiseptic products against EBOV.

### Virucidal activity of PVP-I

Our study confirmed the excellent virucidal activity of PVP-I described in the literature. In one study by Sauerbrei and Wutzler [23], PVP-I solution and PVP-I/alcohol solution showed good efficacy against enveloped viruses (vaccinia virus and bovine viral diarrhoea virus [BVDV]) within 30 s. With the non-enveloped viruses tested, the PVP-I solution showed good efficacy within 5 min against polyomavirus SV40 and adenovirus, but took ≥60 min to inactivate poliovirus type 1. The PVP-I/alcohol solution showed better overall efficacy, especially against the non-enveloped viruses, causing significant inactivation of all test viruses within 5 min, and highlighting the synergistic virucidal activity of PVP-I and alcohol.

In another study by Steinmann et al. [27], a hand wash containing 7.5 % PVP-I was active against vaccinia virus and BVDV within 30 s, but not against the non-enveloped viruses tested. However, in a fingerpad test simulating practical conditions, the PVP-I containing soap was superior to other sanitisers against the non-enveloped viruses.

Although the minimum contact time for hygienic hand rub and hand wash defined in EN14476:2013 [7] is 30 s, we investigated the four PVP-I products with a minimum contact time of 15 s. We did this to study the kinetics of the PVP-I products against MVA over time, and because we assumed that a sufficient degree of effectiveness would not be shown after 15 s. However, all four undiluted preparations efficiently inactivated the European test virus MVA after as little as 15 s.

Protein load (e.g. blood or sputum) may have an influence on the activity of disinfectants, including PVP-I products [28, 29]. In our study, there was a relatively small difference in the virucidal activity of PVP-I when comparing clean and dirty conditions, and then only after 1:100 dilution of the products. Under dirty conditions, a 5-fold higher concentration of PVP-I was needed to inactivate MVA than under clean conditions.

### Limitations

The results of suspension tests only allow the virucidal efficacy of a product to be predicted, and do not give information about its effectiveness in practice. In order to further improve our knowledge of the time/concentration relationships of hand rubs and hand wash products which reflect daily needs in clinical surroundings, more practical testing would be necessary. EN1500 [30] describes a bactericidal test simulating practical conditions in volunteers, however, there is no enveloped harmless virus available that can be used to inoculate the volunteers. More than 30 years ago, tests with vaccinia virus on the fingertips of volunteers were performed [31], however today this can no longer be justified ethically.

### Hand hygiene

Hand hygiene is an infection control procedure with clearly demonstrated efficacy, and remains the cornerstone of efforts to reduce the spread of infection. According to WHO guidelines, hand hygiene is the most important infection prevention and control measure against EBOV [6]. Generally, there are two methods of hand hygiene, social hand washing and hygienic hand disinfection [32, 33]. Socially clean hands are achieved using soap and water, which removes transient microorganisms. Social hand washing should be practised before and after performing routine tasks in all clinical areas. Hygienic hand disinfection is carried out with alcohol-based hand rubs toto remove or destroy all transient microorganisms, and may have a prolonged effect. It is recommended, for example, during outbreaks of infections, and after contact with body fluids or infectious material.

Since the skin is a routine source of infection transmission, the use of PVP-I for hand disinfection may be an alternative to WHO recommended alcohol-based hand rubs. Medicated soaps containing PVP-I are available. Thus, the hygiene measure of hand washing is supported by the virucidal effect of PVP-I. This could be extended beyond surgical use. Healthcare professionals, patients, family members and professionals at risk of infection could use these products to protect themselves after exposure to patients.

PVP-I soap has shown similar efficacy to alcohol-based hand sanitisers against norovirus in a hand wash model [27]. Chlorhexidine- and triclosan-based hand washes, however, were not effective against norovirus in suspension tests and practical application tests [27].

In another study in a neonatal ICU setting, a PVP-I based hand wash showed antimicrobial efficacy comparable to an alcohol-based hand rub, with both being superior to use of soap and water alone [34]. In relation to EBOV and similar viruses, PVP-I has been recommended as a hand disinfectant in laboratories dealing with samples collected from patients with suspected viral haemorrhagic fevers [35].

The results of our study support these findings, and suggest that hand washing with PVP-I based products can help to prevent infection with EBOV. In addition to hand washing, PVP-I preparations may have other applications in reducing the spread of EBOV, as they are not limited to use on intact skin but can also be administered to the mucosa of the mouth, nose, eye, urinary and genital tract [36, 37]. Treatment of these potential entry points for EBOV and other viruses may not be appropriate for other preparations, such as more highly concentrated alcohol-based products.

## Conclusions

All of the PVP-I products tested showed excellent efficacy and fast virucidal activity against both MVA and EBOV after as little as 15 s. The European test virus MVA was even more resistant to PVP-I under dirty conditions than EBOV. Therefore, we consider virucidal tests with MVA a suitable alternative for the claim ‘virucidal active against enveloped viruses for hygienic hand rub and hand wash’.

These results suggest that PVP-I represents an effective measure to help prevent infection and limit the spread of Ebola virus disease. This is helpful information for the implementation of appropriate guidance for people exposed to EBOV, and confirms the excellent virucidal efficacy of PVP-I against enveloped viruses.

## Abbreviations

ATCC: American Type Culture Collection
BHK: baby hamster kidney cells
BSA: Bovine serum albumin
BSL-4: Biosafety level 4
BVDV: Bovine viral diarrhoea virus
CCLV: Collection of Cell Lines in Veterinary Medicine
CDC: Centers for Disease Control and Prevention
D-MEM/MEM: Dulbecco’s Minimum Essential Medium
EBOV: Ebola virus
FCS: Foetal calf serum
ICU: Intensive care unit
LVP: Large volume plating
MERS-Co: Middle East respiratory syndrome coronavirus
MVA: Modified vaccinia virus Ankara
PVP-I: Povidone iodine
SARS: Severe acute respiratory syndrome
TCID_50_/mL: Tissue culture infectious dose 50 %
WHO: World Health Organization
